# Surveillance of Human Rotavirus in Wuhan, China (2011–2019): Predominance of G9P[8] and Emergence of G12

**DOI:** 10.3390/pathogens9100810

**Published:** 2020-10-02

**Authors:** Xuan Zhou, Yuan-Hong Wang, Bei-Bei Pang, Nan Chen, Nobumichi Kobayashi

**Affiliations:** 1 Division of Microbiology, Wuhan Centers for Disease Control and Prevention, Wuhan 430024, China; ice2bhi@gmail.com (X.Z.); pangbei0429@163.com (B.-B.P.); 2 Department of Aquatic Animal Medicine, College of Fisheries, Huazhong Agricultural University, Wuhan 430070, China; chennan@mail.hzau.edu.cn; 3 Department of Hygiene, Sapporo Medical University School of Medicine, Sapporo 060-8556, Japan; nkobayas@sapmed.ac.jp

**Keywords:** genotype, G12, genome, phylogenetic analysis, reassortant

## Abstract

Rotaviruses are a major etiologic agent of gastroenteritis in infants and young children worldwide. To learn the shift of genotypes and genetic characteristics of Rotavirus A (RVA) causing diarrhea in children and adults, a hospital-based surveillance of rotavirus was conducted in Wuhan, China from June 2011 through May 2019, and representative virus strains were phylogenetically analyzed. Among a total of 6733 stool specimens collected from both children and adults with acute gastroenteritis, RVA was detected in 25.5% (1125/4409) and 12.3% (285/2324) of specimens, respectively. G9P[8] was the most common genotype (74.5%), followed by G1P[8] (8.7%), G2P[4] (8.4%), and G3P[8] (7.3%), with G9P[8] increasing rapidly during the study period. The predominant genotype shifted from G1P[8] to G9P[8] in 2012–2013 epidemic season. G12P[6] strain RVA/Human-wt/CHN/Z2761/2019/G12P[6] was detected in April 2019 and assigned to G12-P[6]-I1-R1-C1-M1-A1-N1-T2-E1-H1 genotypes. Phylogenetic analysis revealed that VP7, VP4, VP6, VP3, NSP1, NSP2, and NSP5 genes of Z2761 clustered closely with those of Korean G12P[6] strain CAU_214, showing high nucleotide identities (98.0–98.8%). The NSP3 gene of Z2761 was closely related to those of G2P[4] and G12P[6] rotaviruses in Asia. All the eleven gene segments of Z2761 kept distance from those of cocirculating G9P[8], G1P[8], and G3P[8] strains detected in Wuhan during this study period. This is the first identification of G12 rotavirus in China. It is deduced that Z2761 is a reassortant having DS-1-like NSP3 gene in the background of G12P[6] rotavirus genetically close to CAU_214.

## 1. Introduction

Group A rotavirus (RVA) is one of the leading etiological agents of diarrheal disease among children, and more than 120,000 deaths among children younger than five years were due to rotavirus in 2016, the fifth most fatal pathogen globally [1]. Vaccination is an essential strategy for the prevention and control severe diseases caused by rotavirus infection. Two rotavirus vaccines (Rotarix^TM^ and RotaTeq^®^) are recommended for routine immunization of all infants by the World Health Organization (WHO) and have been introduced in more than 100 countries except for China [2,3,4]. A third rotavirus vaccine (Lanzhou Lamb rotavirus vaccine (LLR)) derived from a lamb G10P[12] rotavirus strain was licensed in 2000 and available in mainland of China [5]. Children in China were inoculated about 60 million doses of LLR during 2008–2014 [6].

Rotavirus, a member of the family *Reoviridae*, is a nonenveloped and double-stranded RNA virus that contains a segmented genome, encoding six structural proteins (VP7, VP4, VP6, VP1-VP3) and six nonstructural proteins (NSP1-NSP6). The two outer capsid proteins VP7 and VP4 carrying neutralization-specific epitopes are determinants of G and P genotypes of rotavirus, respectively, which represent the dual-nomenclature system [7,8]. Thirty-six G and fifty-one P types have been differentiated so far [9]. Among them, at least 12 G and 15 P types, and over 60 G-P combinations have been reported in human group A rotavirus [10,11]. G1P[8], G2P[4], G3P[8], G4P[8], and G9P[8] are the common G-P combinations in human rotaviruses and responsible for over 80% of the circulating genotypes globally [12,13].

An extended classification and nomenclature of RVA have been established to define genotypes for 11 segments of whole genome by the Rotavirus Classification Working Group (RCWG). The nomenclature Gx-P[x]-Ix-Rx-Cx-Mx-Ax-Nx-Tx-Ex-Hx represents genotypes of the VP7-VP4-VP6-VP1-VP2-VP3-NSP1-NSP2-NSP3-NSP4-NSP5-encoding gene segments, respectively, with “x” indicating genotype numbers [10]. Currently, partial or complete sequences of all 11 gene segments of RVA strains are used to analyze and compare with those of other strains to determinate genetic relationships [14,15,16,17].

Human G12 rotavirus was first detected in the Philippines in 1987, and no further cases had been reported until 1998 [18]. Strain se585 was reported in the USA in the 1990s [19], and then G12P [9] strain Arg720 was detected in Argentina in 1999 [20]. G12 rotavirus spread and circulated in Asian countries from 2002 through 2008 [21,22,23,24,25,26,27,28,29,30,31,32,33]. In the last decade, prevalence of G12-P[8]/P[6] human rotavirus has increased globally and was reported to be predominant in USA [34], Nigeria [35], St. Louis [36], Nepal [37], Brazil [38], Bangladesh [39], and Mozambique [40]. G12 is now recognized as the sixth epidemiological important genotype associated with infections in humans [13,41,42]. Although G12 rotavirus was introduced into Asia in the early 2000s and was prevalent in the countries and regions adjacent to China, it has never been reported in China.

A constant surveillance on human rotavirus in all age groups with acute gastroenteritis has been conducted since 2000 in Wuhan, China [43,44,45]. The aims of the present study were to reveal the prevalence and shifting of human RVA genotypes and to understand the origin and molecular evolution of the emerging G12 strain based on its full genome analysis.

## 2. Results

### 2.1. Detection of Rotavirus, VP7 and VP4 Genotyping

**Figure 1:**
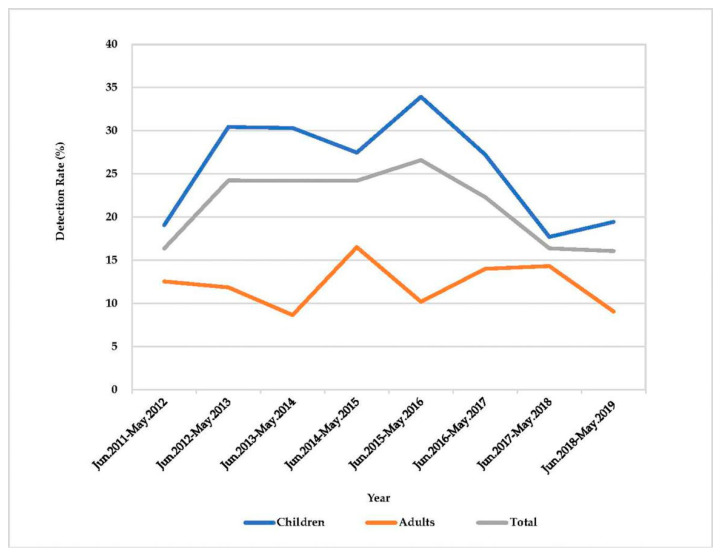
Detection rate (%) of Rotavirus A (RVA) in children and adults in Wuhan between 2011 and 2019.

**Figure 2:**
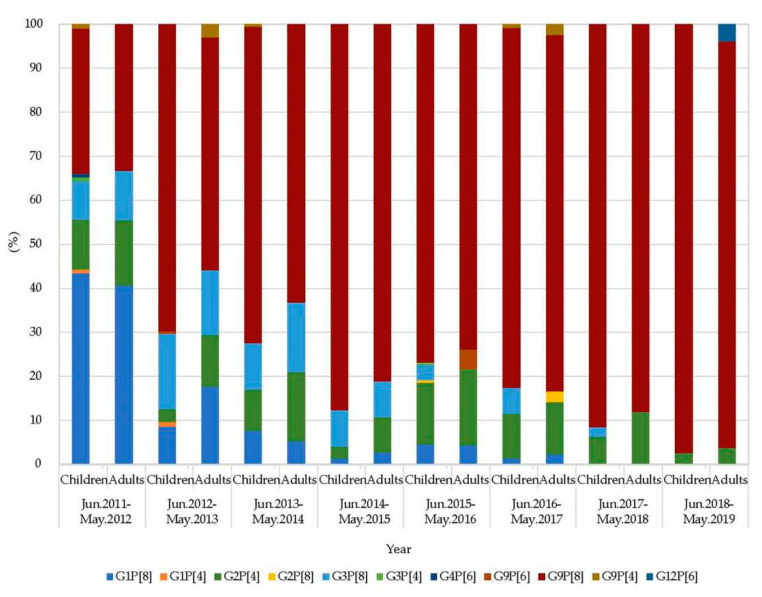
Composition ratio (%) of G- and P- genotypes of RVA detected in children and adults in Wuhan between 2011 and 2019.

A total of 6733 fecal specimens from 4409 children (under 15 years old) and 2324 adults (15–96 years old) were collected from June 2011 through May 2019. RVA was detected in 25.5% (1125/4409) and 12.3% (285/2324) of specimens from children and adults, respectively (Figure 1). Rotavirus B and C were not detected. A total of 11 G-P combinations were determined throughout the study period. Generally, G9P[8] was predominant (74.5%), followed by G1P[8] (8.7%), G2P[4] (8.4%), and G3P[8] (7.3%) (Table S1, Figure 2). Of these, G9P[8] was predominant both in children (75.8%) and adults (69.1%), respectively, followed by G1P[8] (8.0%), G3P[8] (7.6%), and G2P[4] (7.6%) in children, and G2P[4] (11.9%), G1P[8] (11.2%), and G3P[8] (6.0%) in adults.

During the study period, G9P[8] was predominant except for the epidemic season 2011–2012 when G1P[8] was more frequent than the other genotypes. G9P[8] was more common in children than in adults (minimum χ^2^ = 10.81, *p* < 0.01) during this study period except for June 2011 to May 2012 and June 2017 to May 2018. In the present study, G12 rotavirus strain RVA/Human-wt/CHN/Z2761/2019/G12P[6] was detected in April 2019 (Figure S1).

The detection rate was 25.5% (1119/4385) in children with exact age information. The highest detection rate was 40.0% (351/877) in 13–24-month age group, followed by 28.4% (537/1888), 19.8% (60/303), 13.3% (148/1109), and 11.1% (23/208) in 7–12, 25–60, 0-6-month age groups and 5-14-year age group, respectively. G9P[8] was the most commonly detected genotype in different age groups, followed by G2P[4] in 25m–14y age group, G1P[8] in 0–12m age group, and G3P[8] in 13–24 age group (Table S2).

### 2.2. The Case of G12 RVA Infection

The patient infected with G12P[6] RVA was a 59-year-old women who retired and was living in Jianghan District of Wuhan city. On early onset of gastroenteritis, the patient had diarrhea four times a day without vomiting or fevers. Then she visited the outpatient department of Wuhan Commercial Staff Hospital on 3 July 2019. After treatment with oral rehydration salts within three days, she recovered. The patient had not been vaccinated of LLR and none of the family members developed symptoms of gastroenteritis. She did not have any pets. Before the occurrence of diarrhea, she had neither gone out for tourism nor contacted with wild animals.

### 2.3. Genotype Constellation

The whole genomes were determined for the G12 strain Z2761 and six simultaneously cocirculating G9P[8], G1P[8], and G3P[8] strains (Table 1). Genotype of strain Z2761 was assigned to G12-P[6]-I1-R1-C1-M1-A1-N1-T2-E1-H1. All the G9P[8], G1P[8], and G3P[8] strains showed genetic constellation of Wa-like human rotavirus. Among these strains, nucleotide sequence identities of G9-VP7 genes and P[8]-VP4 genes were 99.7–100.0% and 98.4–99.9%, respectively. Among these P[8] strains, nucleotide identities of the VP1, VP2, VP3, VP4, VP6, NSP2, and NSP5 genes were over 97.9%, whereas NSP1, NSP3, and NSP4 genes showed lower identities with minimum value of 81.0%, 94.3%, and 79.9%, respectively.

### 2.4. Phylogenetic Analysis

#### 2.4.1. Structural Protein Genes

**Figure 3:**
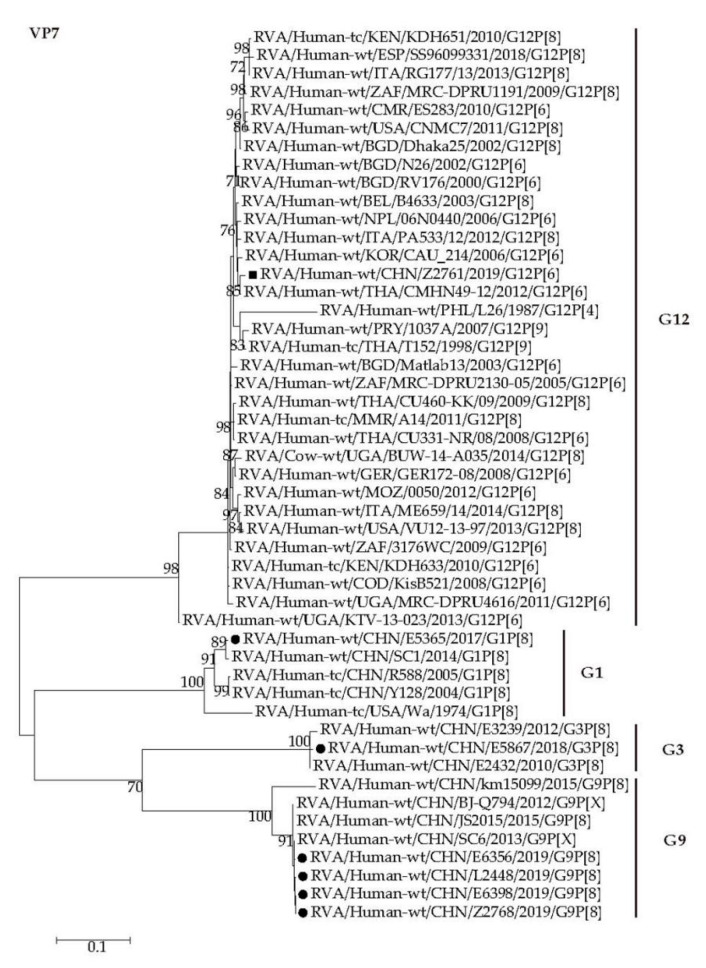
Phylogenetic dendrogram based on complete coding regions of the VP7 genes of representative RVAs. The best nucleotide substitution model is T92 + G. Bootstrap values below 70% are not shown. The G12P[6] strain Z2761 (in boldface) is highlighted with filled square. The contemporaneous G1, G3, and G9 strains are highlighted with filled circles.

Phylogenetically, the VP7 gene of Z2761 was grouped into G12 RVA strains and clustered with those of strains detected in neighboring countries including Korea (CAU_214), Thailand (CHMN49-12) and Nepal (06N0440) with the minimum nucleotide identity of 98.4% and other human G12 strains reported worldwide such as in Europe, Africa, and the USA, showing nucleotide identity over 96.5% (Figure 3).

**Figure 4:**
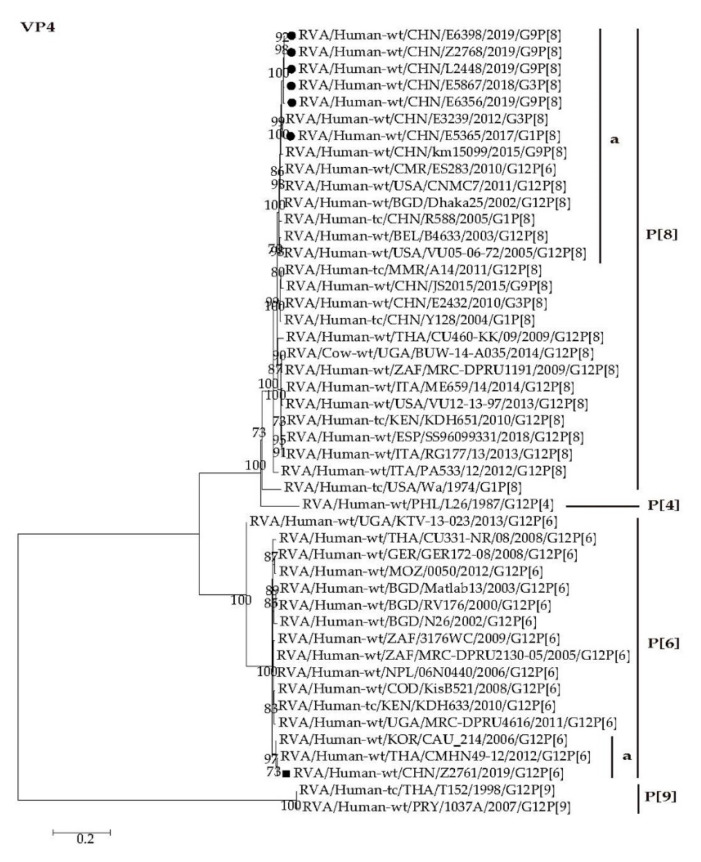
Phylogenetic dendrogram based on complete coding regions of the VP4 genes of representative RVAs. The best nucleotide substitution model is HKY + G + I. Bootstrap values below 70% are not shown. The G12P[6] strain Z2761 (in boldface) is highlighted with filled square. The contemporaneous G1, G3, and G9 strains are highlighted with filled circles.

The VP4 gene of G12P[6] strain Z2761 clustered closely with those of CAU_214 and CMHN49-12 with the nucleotide identities of 98.8% and 99.1%, respectively (Figure 4, P[6]a). The P[8]-VP4 genes of E5365, E5867, E6356, E6398, Z2678, and L2448 were closely related to those of the human strains circulating in China (E3239 and km15099) with minimum nucleotide identity of 98.4% (Figure 4, P[8]a).

**Figure 5:**
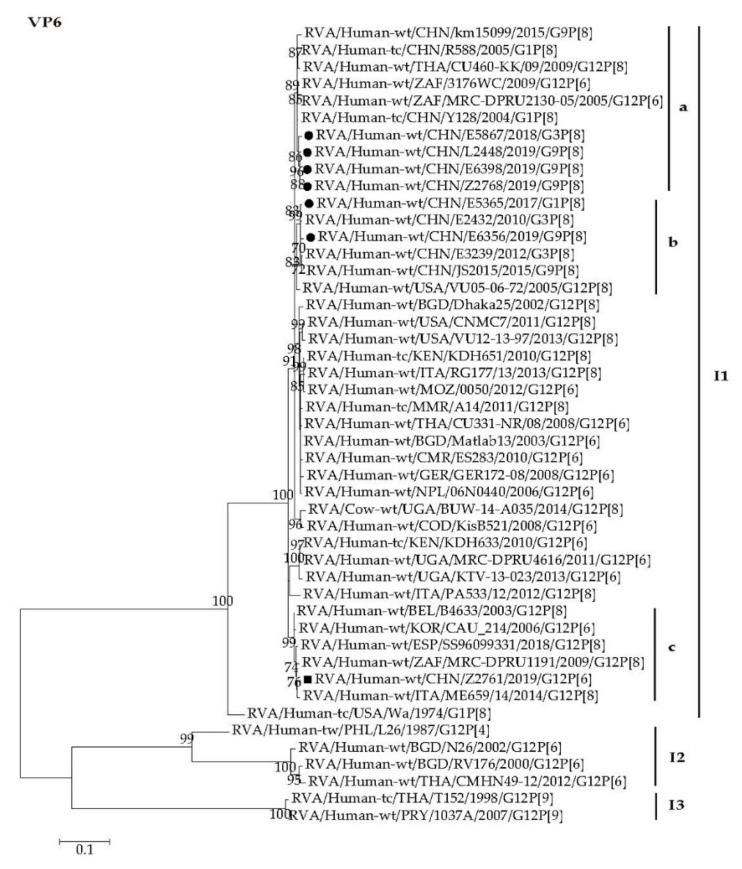
Phylogenetic dendrogram based on complete coding regions of the VP6 genes of representative RVAs. The best nucleotide substitution model is T92 + G + I. Bootstrap values below 70% are not shown. The G12P[6] strain Z2761 (in boldface) are highlighted with filled square. The contemporaneous G1, G3, and G9 strains are highlighted with filled circles.

The VP6 genes of all the seven strains were assigned to the I1 genotype. Z2761 VP6 gene clustered closely with those of the strains from Korea (CAU_214), South Africa (MRC-DPRU1191), and European countries including Italy (ME659), Belgium (B4633), and Spain (SS96099331) with the minimum nucleotide identity of 98.7% (Figure 5, I1c). The VP6 genes of E5365 and E6356 were close to those of Chinese strains (E2432, E3239, and JS2015) with minimum nucleotide identity of 99.0% (Figure 5, I1b). The VP6 genes of E5867, E6398, L2448, and Z2768 were close to those of Chinese strains (R588 and Y128) and South African strains (3176WC and MRC-DPRU2130-05) with minimum nucleotide identity of 99.0% (Figure 5, I1a).

The VP1 and VP2 genes of all the seven strains were assigned to the R1 and C1 genotypes, respectively (Figure S2 and Figure S3). The VP1 and VP2 genes of Z2761 clustered closely with those of European strains (RG177 and ss96099331) and African strains (KDH651 and 0050) with high nucleotide identity of 97.5–98.7% (Figure S2, R1b and Figure S3, C1a). The *VP1* genes of E5365, E5867, E6356, E6398, L2448, and Z2768 were close to those of strains from China and Thailand (JS2015, E3239, CU331-NR, and CU460-KK), with high nucleotide identities of 98.7–99.8% (Figure S2, R1a). Similarly, the VP2 genes of the six strains mentioned above were close to those of Chinese and South African strains (Y128, R588, km15099, JS2015, E2432, and MRC-DPRU2130-05), with minimum nucleotide identity of 98.3% (Figure S3, C1b).

The VP3 genes of all the seven strains were grouped into the M1 genotype (Figure S4). The VP3 gene of Z2761 clustered closely with those of the strains from Korea (CAU_214) and Italy (ME659) with nucleotide identity over 98.1% (Figure S4, M1a). The VP3 genes of E5365, E5867, E6356, E6398, L2448, and Z2768 were close to those of Chinese strains (R588, Y128, E2432, E3239, and JS2015) with minimum nucleotide identity of 98.1% (Figure S4, M1b).

#### 2.4.2. Nonstructural Protein Genes

The NSP1 genes of all seven strains were assigned to the A1 genotype (Figure S5). The NSP1 gene of Z2761 was close to that of Korean strain CAU_214 showing nucleotide identity of 98.7% (Figure S5, A1b). The NSP1 genes of E5365, E5867, E6356, E6398, L2448, and Z2768 were assigned to different lineages (Figure S5). The NSP1 gene of E5867 clustered with that of Chinese G3P [8] strain E2432 showing high nucleotide identity (98.9%) (Figure S5, A1c). The NSP1 gene of E5365 clustered closely with those of Chinese G1P[8] strains (R588 and Y128) with high nucleotide identity (98.7%) (Figure S5, A1d). The NSP1 genes of E6356, E6398, L2448, and Z2768 clustered closely to those of Chinese G9P[8] strains (JS2015 and km15099) with high nucleotide identities of 97.7–98.6% (Figure S5, A1a).

The NSP2 and NSP5 genes of all the seven strains were assigned to the N1 and H1 genotypes, respectively (Figures S6 and S7). The NSP2 and NSP5 genes of Z2761 were distant from those of cocirculating G9P[8], G1P[8], and G3P[8] strains from Wuhan in this study period, showing maximum nucleotide identities of 97.5% (Figure S6, N1b; Figure S7, H1b). The NSP2 genes of E5365, E5867, E6356, E6398, L2448, and Z2768 clustered closely with those of Chinese strains (R588, Y128, E2432, E3239, km15099, and JS2015) and a South African strain, showing high nucleotide identities of 98.0–99.5% (Figure S6, N1a). The NSP5 genes of E5365 and E6356 were genetically close to those of Asian strains from China and Korea, and western country strains from Belgium and the USA, showing high nucleotide identities of 98.3–99.5% (Figure S7, H1c). The NSP5 genes of E5867, E6398, L2448, and Z2768 were closely related to those of human strains and a bovine strain (BUW-14-A035) from Asia, Europe, Africa, and North America, showing high nucleotide identities of 98.0–99.3% (Figure S7, H1a).

**Figure 6:**
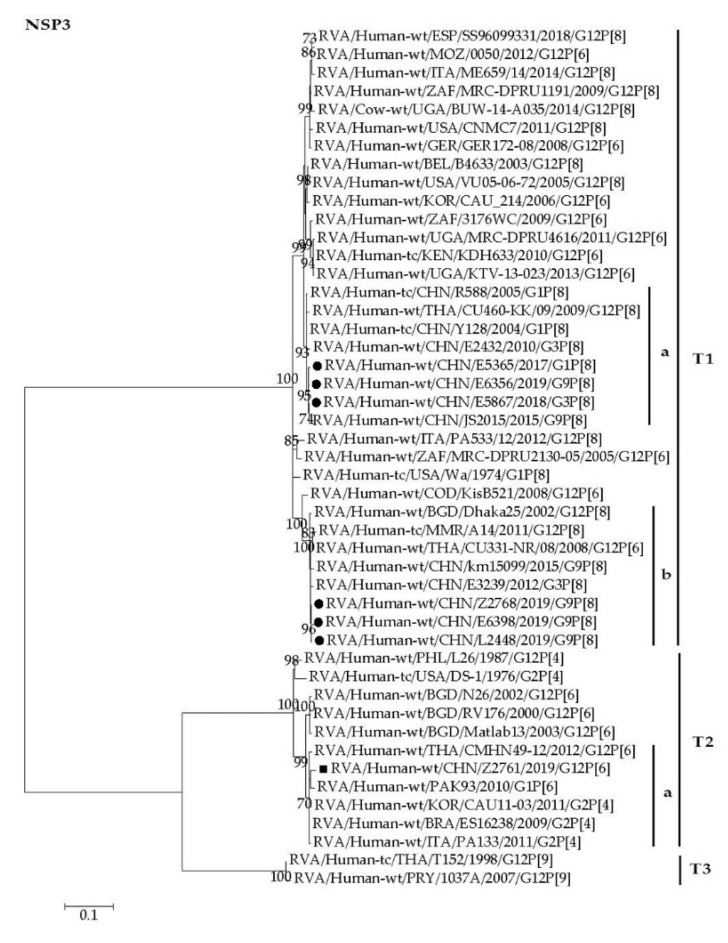
Phylogenetic dendrogram based on complete coding regions of the NSP3 genes of representative RVAs. The best nucleotide substitution model is T92 + I. Bootstrap values below 70% are not shown. The G12P[6] strain Z2761 (in boldface) are highlighted with filled square. The contemporaneous G1, G3, and G9 strains are highlighted with filled circles.

The NSP3 gene of Z2761 was assigned to the T2 genotype clustering closely with strains from Asian countries (CMHN49-12, PAK93, and CAU11-03) and western countries (ES16238 and PA133), showing minimum nucleotide identities of 98.2% (Figure 6, T2a). The NSP3 genes of E6398, L2448, Z2768, E5365, E5867, and E6356 were assigned to the T1 genotype and grouped into different lineages. The NSP3 genes of E6398, L2448, and Z2768 clustered closely with those of Asian strains from China, Bangladesh, Thailand, and Burma, showing minimum nucleotide identity of 99.0% (Figure 6, T1b). The NSP3 genes of E5365, E5867, and E6356 clustered with those of strains from China and Thailand with high nucleotide identities of 98.5–99.4% (Figure 6, T1a).

**Figure 7:**
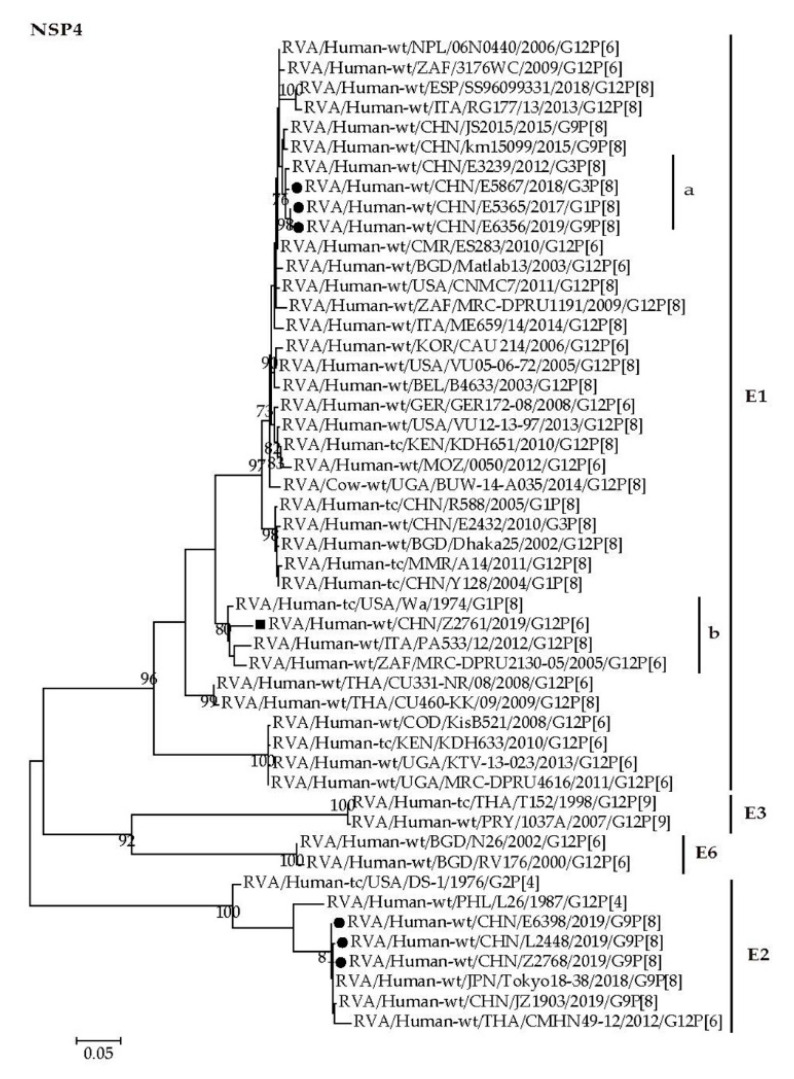
Phylogenetic dendrogram based on complete coding regions of the NSP4 genes of representative RVAs. The best nucleotide substitution model is T92 + G. Bootstrap values below 70% are not shown. The G12P[6] strain Z2761 (in boldface) are highlighted with filled square. The contemporaneous G1, G3, and G9 strains are highlighted with filled circles.

The NSP4 genes of Z2761, E5365, E5867, and E6356 were assigned to the E1 genotype (Figure 7, E1). The NSP4 gene of Z2761 clustered with those of South Africa strain MRC-DUPR2130-05, Italian strain PA533 and the USA strain Wa, showing nucleotide identities of 95.3–97.0% (Figure 7, E1b). The NSP4 genes of E5365, E5867, and E6356 were close with those of Chinese strains with high nucleotide identities of 98.5–99.2% (Figure 7, E1a). The NSP4 genes of three G9P[8] strains, E6398, L2448, and Z2768, were assigned to the E2 genotype that included those of strains from China and neighboring countries (JZ1903, Tokyo18-38, and CMHN49-12) with high nucleotide identity (97.7–100%) (Figure 7, E2).

## 3. Discussion

Comparing to the previous surveillance on rotavirus in Wuhan, the detection rate of RVA increased in adults, while being stable in children (χ^2^ = 13.2, *p* < 0.01), and the rate was higher in children rather than in adults repeatedly [43,44,45]. When the data from other countries were compared, lower incidence (25.5%) of RVA in children was observed in Wuhan except for that in 13–24 month age group (40.0%), similarly in proportion to that reported in Southeast Asia (40.78%) [46,47]. In present study, major of specimens were collected from outpatients and most cases with relatively mild symptoms, which could be responsible for the lower incidence of RVA in children. Considering the highest incidence was in the 6–12-month age group, early introduction of routine rotavirus vaccination to children is desirable.

The incidence of rotavirus diarrhea in adults has been reported ranging from 2 to 22% of gastroenteritis cases in numerous countries [48,49]. In the present study, the incidence (12.3%) of RVA was higher than that in Thai adults (8.7%) [50]. It is suggested that rotavirus gastroenteritis in adult population should be also prevented by any intervention in China.

G1/G3/G9-P[8] and G2P [4] were most commonly reported in the epidemiological and clinical studies of rotavirus in China from 1994 to 2013 [51]. The genotype distribution of RVA has been varying over the years. The predominant strain of RVA has shifted from G1P[8] and G3P[8] to G9P[8], with remarkably increased proportion of G9P[8] from 3.4% in 2009 to 60.9 % in 2015 in China [52]. G3P[8] was predominant during 2000–2008 in Wuhan and the predominant genotype G3 shifted to G1 in 2011 [43,44,45]. G9P[8] was the most common genotype combination both in children and adults and has been predominant since 2012 in present study period. Simultaneously, the predominant G9P[8] was detected in other provinces and cities in China, such as Beijing [53,54], Gansu [53], Jiangsu [55], Shanghai [56], Henan [57], Kunming [58], Fujian [59], and Chongqing [60]. The predominant RV genotypes circulating all over the world are G1P[8], G2P[4], G3P[8], G4P[8], and G9P[8], while G12P[6] and G12P[8] are emerging genotypes [12,13,61]. The distribution of genotypes of RVA in Wuhan between 2011 and 2019 was consistent with the global prevalence of RVA with different genotypes, particularly with that in Southeast Asian countries [47].

Rotavirus vaccination is not included in China’s national immunization program. The population of children under five years of age is about 300,000 in Wuhan. Of these, 3,000–25,000 infants received the vaccination annually since the year of 2005 when LLR had been introduced. Low coverage rates of vaccine may be responsible for the ongoing epidemics in Wuhan. The detection rate in children did not decrease after vaccination [43,44,45]. It was deduced that the influence of LLR on the prevalence and genotype of RVA in Wuhan was limited.

Whole genomic phylogenetic analysis showed those G1/G3/G9-P[8] RVAs determined in this study were close to Wa genogroup RVA G1/G3P[8] strains (R588, Y128, E3239 and E2432, etc.) detected in Wuhan [62,63] and G9P[8] strains (BJ-Q794, km15099, SC6 and JS2015, etc.) obtained in Beijing, Kunming [64], Chengdu [65], and Jiangsu [55] previously (Figure 3, Figure 4, Figure 5, Figure 6 and Figure 7 and Figures S2–S7). It is notable that three G9P[8] strains are reassortants possessing DS-1 like NSP4 genes in the genetic background of Wa-like rotavirus (Figure 7, E2). The reassortant G9-P[8]-I1-R1-C1-M1-A1-N1-T1-E2-H1 were also detected in Jinzhou, China, and Japan [66,67]. Since the identification of NSP4 genotype is not included in the routine surveillance, it is not clear when this kind of reassortant appeared in Wuhan. It is worth tracing its origin and proportion in all RVAs.

Phylogenetically, the VP7, VP4, VP6, VP3, NSP1, NSP2, and NSP5 genes of the G12P[6] strain Z2761 were close to those of the Korean G12P[6] strain (CAU_214), while all the structural and nonstructural protein genes of Z2761 kept distance from those cocirculating strains in Wuhan in this study period. Because of the limited epidemical information, the origin of infection and the route of transmission of Z2761 was not clear. Considering the phylogenetic characters of the whole genome of Z2761 and case information of the patient, this emerging G12P[6] RVA in China was hypothesized to be brought from rotavirus sharing a common ancestor with strain CAU_214. During transmission of G12P[6] RVA, NSP3 gene of DS-1-like human rotavirus was suggested to be reassorted via coinfection, resulting in the emergence of strain Z2761. To our knowledge, Z2761 is the first G12 strain detected in China. More information should be accumulated through intensive nationwide surveillance to monitor prevalence of G12 in China.

## 4. Materials and Methods

### 4.1. Specimens

A hospital-based surveillance of sporadic rotavirus diarrhea was conducted in Wuhan, China. Fecal specimens were collected from inpatients and outpatients in four hospitals (Renming Hospital of Wuhan University, Wuhan Commercial Staff Hospital, Wuhan the Sixth Hospital, and Wuhan Children’s Hospital) from June 2011 through May 2019. All the specimens were stored at −80 °C. Fecal specimens were analyzed as part of routine surveillance of infectious diseases that had been approved by the ethics committee of Wuhan Centers for Disease Control and Prevention. Oral informed consent was obtained from the patients or guardians for all samples collected.

### 4.2. Investigation on The Case Infected G12 Rotavirus

Epidemiological information including the demographics, symptoms, and contact history was collected in the hospital. More information of the case infected G12 RVA was obtained by a telephone survey.

### 4.3. Detection of Rotavirus

Viral dsRNA was extracted from 400 microliters of 10% stool suspension with sodium dodecyl sulfate (SDS) and phenol and precipitated with ethanol [68]. RNA segments of rotavirus were separated by polyacrylamide gel electrophoresis (PAGE) and stained with silver nitrate [69].

### 4.4. Genotyping of RVA

Viral dsRNA was extracted from 200 microliters of 10% stool suspension by using the automatic nucleic acid extraction system NP968S with Nucleic Acid Extraction Kit (Jiangsu Tianlong Science and Technology Co. Ltd., Jiangsu, China) according to the manufacturer’s instructions. Rotavirus G-type and P-type had been determined by nested reverse transcription-polymerase chain reaction (RT-PCR) from 2011 through 2015 [70,71,72,73].

The genotypes of VP7 and VP4 genes have been determined by analyzing the sequences of the first round PCR products since 2016 [70,71,72,73]. The nucleotide sequences of primers were listed in Table S3. PCR products were subjected to direct sequencing by Sanger method at Sangon Biotech (Shanghai) Co. Ltd. (Wuhan). The genotype of RVA was preliminarily assigned by the Basic Local Alignment Search Tool (BLAST).

### 4.5. Whole Genome Sequencing

The complete 11 segmented genes of whole genome were amplified by RT-PCR. The nucleotide sequences of primers were listed in Table S3 and included those reported in our previous study [63]. PCR products were subjected to direct sequencing by Sanger method.

### 4.6. Phylogenetic Analysis

Nucleotide sequence of each segment was assembled and edited by DNAMAN software. Genotype was determined by RotaC version 2.0, an online automated genotyping tool for RVAs [74]. The sequence identities were analyzed by Lasergene bio-information software (DNASTAR, Inc, Madison, Wisconsin, USA). Multiple alignments of the sequences were performed using MAFFT v7.471 (Kazutaka Katoh, Osaka, Japan) [75]. Phylogenetic analysis was conducted together with reference strain sequences obtained in the National Center for Biotechnology Information (NCBI) database by MEGA program version 7.1 [76]. The evolutionary history was inferred by using the Maximum Likelihood method, with 1000 bootstrap replicates, based on the best nucleotide substitution model with the lowest Bayesian information criterion (BIC) score in MEGA v7.1 [77]. Initial tree for the heuristic search was obtained automatically by applying Neighbor-Joining and BioNJ algorithms to a matrix of pairwise distances estimated using the Maximum Composite Likelihood (MCL) approach, and then selecting the topology with superior log likelihood value. The tree was drawn to scale, with branch lengths represented by the number of substitutions per site.

### 4.7. Statistical Analysis

Statistical analysis was performed by SPSS version 18.0 (SPSS Inc., Chicago, IL, USA) software. Testing for the statistical significance was performed using the chi-square test. *p*-value less than 0.05 is considered significant.

### 4.8. Accession Numbers of Nucleotide Sequences in Genbank

The sequences of the genomes of strains including RVA/Human-wt/CHN/Z2761/2019/G12P[6], RVA/Human-wt/CHN/E5365/2017/G1P[8], RVA/Human-wt/CHN/E5867/2018/G3P[8], RVA/Human-wt/CHN/E6356/2019/G9P[8], RVA/Human-wt/CHN/E6398/2019/G9P[8], RVA/Human-wt/CHN/L2448/2019/G9P[8], and RVA/Human-wt/CHN/Z2768/2019/G9P[8] were deposited in the GenBank database under accession numbers MN106111–MN106187.

## Figures and Tables

**Table 1:** The complete genotype constellations of G1, G3, G9, and G12 RVAs collected in Wuhan.

Strains	Collection Date (YYYYMM)	Gender (F/M)	Age (Y/M/D)	Genotype ^1^
				VP7-VP4-VP6-VP1-VP2-VP3-NSP1-NSP2-NSP3-NSP4-NSP5 (Genbank Accession Numbers)
RVA/Human-tc/USA/Wa/1974/G1P[8]	1974	-	-	G1-P[8]-I1-R1-C1-M1-A1-N1-T1-E1-H1
				(JX406747-JX406757)
RVA/Human-tc/USA/DS-1/1976/G2P[4]	1976	-	-	G2-P[4]-I2-R2-C2-M2-A2-N2-T2-E2-H2
				(HQ650116-HQ650126)
RVA/Human-wt/CHN/Z2761/2019/G12P[6]	20190307	F	59y	G12-P[6]-I1-R1-C1-M1-A1-N1-T2-E1-H1
				(MN106117, MN106124, MN106131, MN106138, MN106145, MN106152, MN106159, MN106166, MN106173, MN106180, MN106187)
RVA/Human-wt/CHN/E5365/2017/G1P[8]	20170306	M	9m21d	G1-P[8]-I1-R1-C1-M1-A1-N1-T1-E1-H1
				(MN106111, MN106118, MN106125, MN106132, MN106139, MN106146, MN106153, MN106160, MN106167, MN106174, MN106181)
RVA/Human-wt/CHN/E5867/2018/G3P[8]	20180403	M	5m25d	G3-P[8]-I1-R1-C1-M1-A1-N1-T1-E1-H1
				(MN106112, MN106119, MN106126, MN106133, MN106140, MN106147, MN106154, MN106161, MN106168, MN106175, MN106182)
RVA/Human-wt/CHN/E6356/2019/G9P[8]	20190118	M	1y7m	G9-P[8]-I1-R1-C1-M1-A1-N1-T1-E1-H1
				(MN106113, MN106120, MN106127, MN106134, MN106141, MN106148, MN106155, MN106162, MN106169, MN106176, MN106183)
RVA/Human-wt/CHN/E6398/2019/G9P[8]	20190308	M	1y8m	G9-P[8]-I1-R1-C1-M1-A1-N1-T1-E2-H1
				(MN106114, MN106121, MN106128, MN106135, MN106142, MN106149, MN106156, MN106163, MN106170, MN106177, MN106184)
RVA/Human-wt/CHN/L2448/2019/G9P[8]	20190218	F	30y	G9-P[8]-I1-R1-C1-M1-A1-N1-T1-E2-H1
				(MN106115, MN106122, MN106129, MN106136, MN106143, MN106150, MN106157, MN106164, MN106171, MN106178, MN106185)
RVA/Human-wt/CHN/Z2768/2019/G9P[8]	20190318	F	55y	G9-P[8]-I1-R1-C1-M1-A1-N1-T1-E2-H1
				(MN106116, MN106123, MN106130, MN106137, MN106144, MN106151, MN106158, MN106165, MN106172, MN106179, MN106186)

^1^ Genotypes of Wa- and DS-1-like human rotaviruses (prototype strains Wa and DS-1) are shown in green and red, respectively. G12 and P[6] are highlighted with orange font color. -, information not available.

## Supplementary Materials

The following are available online at https://www.mdpi.com/2076-0817/9/10/810/s1, Table S1: Frequency of G- and P- genotypes of RVA detected in children and adults in Wuhan between 2011 and 2019. Table S2: Frequency of G- and P- genotypes of RVA detected in children in Wuhan between 2011 and 2019. Table S3: Primers used for RT-PCR and sequencing in this study. Figure S1: RNA patterns of G12P[6] rotavirus strain (Z2761) and the other six G9P[8] strains collected in March 2019. Lane 1, E6390; Lane 2, E6392; Lane 3, E6394; Lane 4, E6397; Lane 5, E6398; Lane 6, Z2757; Lane 7, Z2761. Figure S2: Phylogenetic dendrogram based on complete coding regions of the VP1 genes of representative RVAs. The best nucleotide substitution model is TN93 + G + I. Figure S3: Phylogenetic dendrogram based on complete coding regions of the VP2 genes of representative RVAs. The best nucleotide substitution model is TN93 + G + I. Figure S4: Phylogenetic dendrogram based on complete coding regions of the VP3 genes of representative RVAs. The best nucleotide substitution model is GTR + G + I. Figure S5: Phylogenetic dendrogram based on complete coding regions of the NSP1 genes of representative RVAs. The best nucleotide substitution model is GTR + G + I. Figure S6: Phylogenetic dendrogram based on complete coding regions of the NSP2 genes of representative RVAs. The best nucleotide substitution model is T92 + I. Figure S7: Phylogenetic dendrogram based on complete coding regions of the NSP5 genes of representative RVAs. The best nucleotide substitution model is T92 + G.
