# Supplementary material for: Surveillance of Human Rotavirus in Wuhan, China (2011–2019): Predominance of G9P[8] and Emergence of G12
Source: Pathogens. 2020 Oct 2;9(10):810. doi: 10.3390/pathogens9100810 (PMC7600066; doi:10.3390/pathogens9100810)
Supplement: Supplementary file 1 [file pathogens-09-00810-s001.zip › Supplementary Materials/Figure S1.pdf]

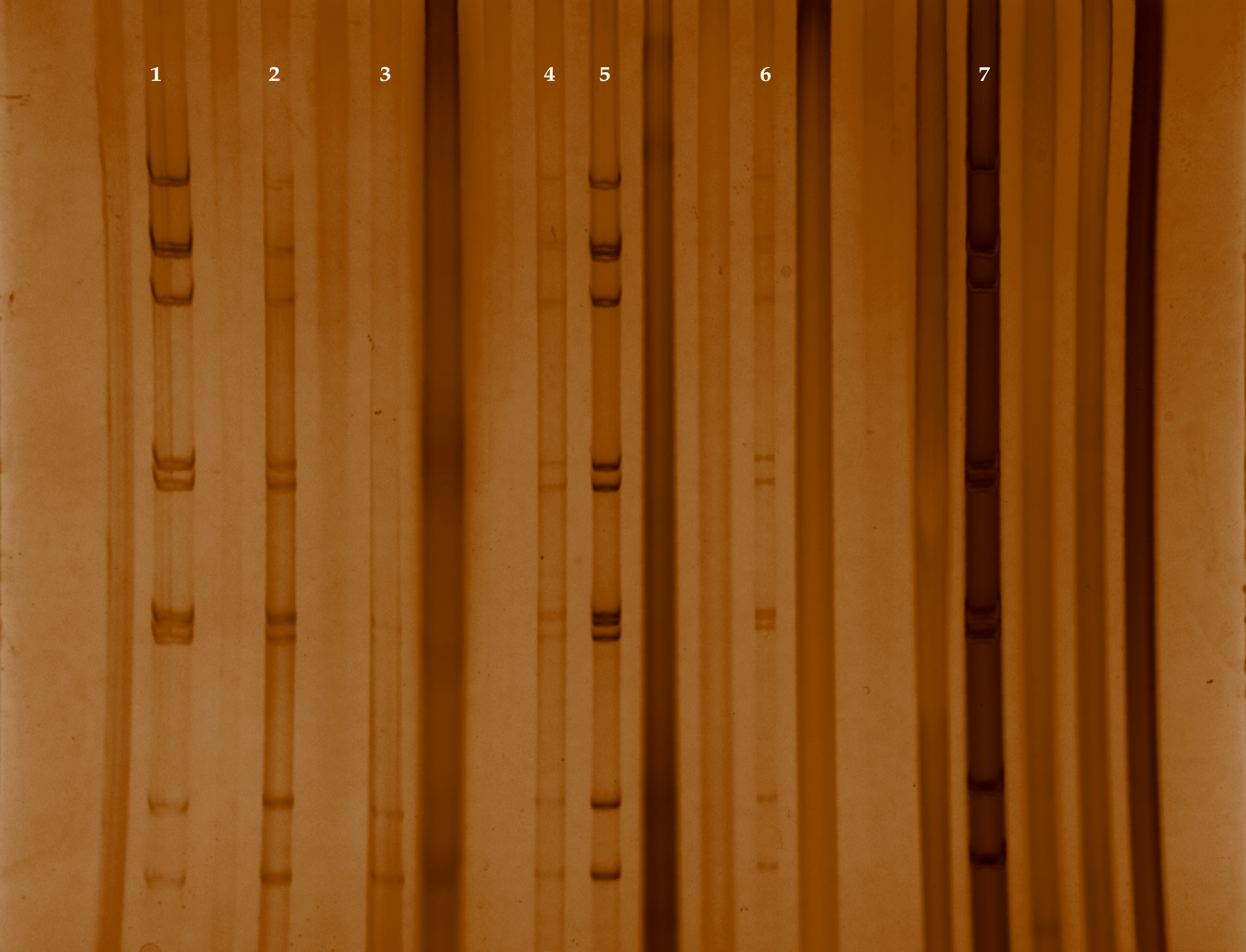
1
2
3
4
5
6
7
