# Supplementary figures and images for: Surveillance of Human Rotavirus in Wuhan, China (2011–2019): Predominance of G9P[8] and Emergence of G12
Source: Pathogens. 2020 Oct 2;9(10):810. doi: 10.3390/pathogens9100810 (PMC7600066; doi:10.3390/pathogens9100810)

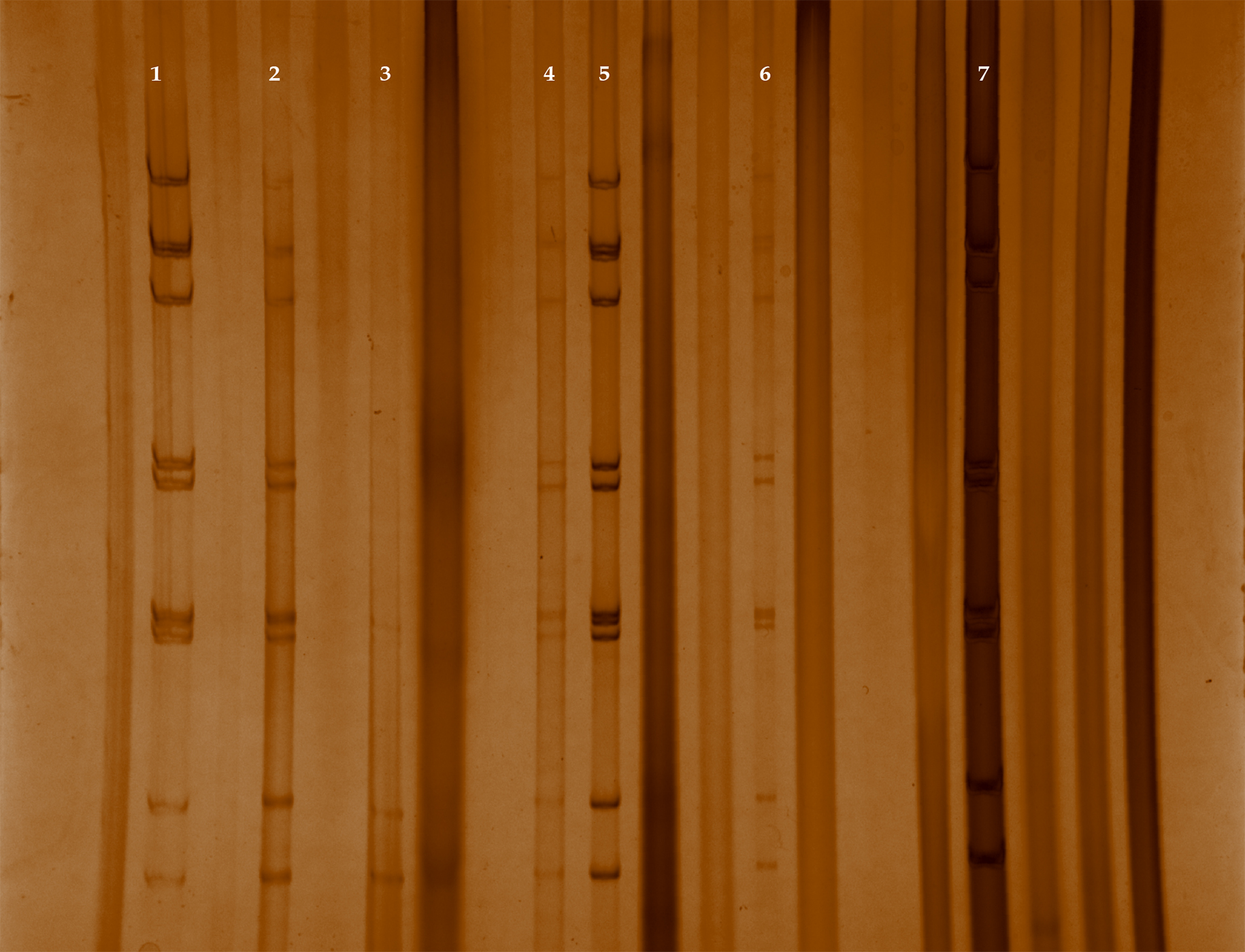

Supplement: Supplementary file 1 [file pathogens-09-00810-s001.zip › Supplementary Materials/Figure S1.jpg]

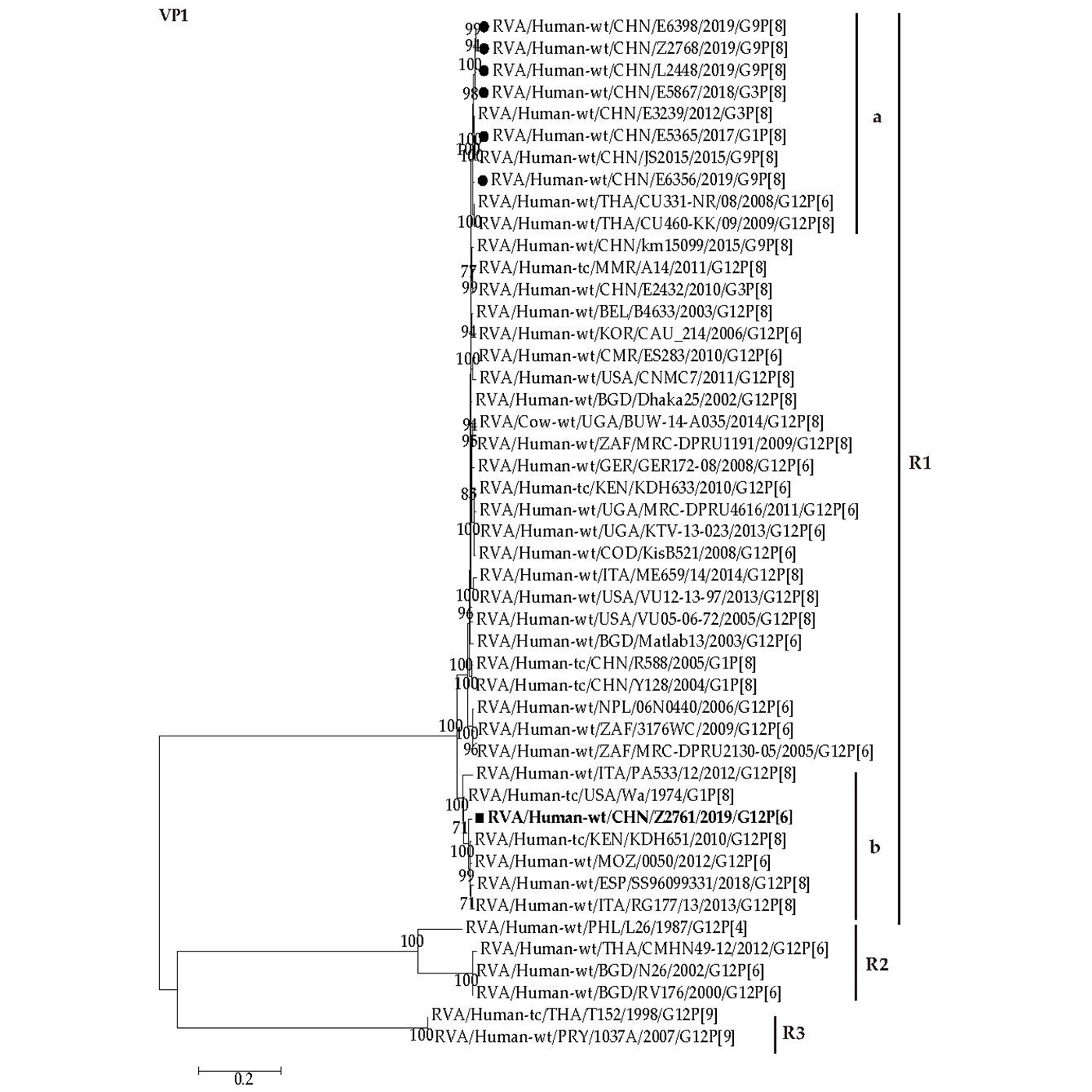

Supplement: Supplementary file 1 [file pathogens-09-00810-s001.zip › Supplementary Materials/Figure S2.jpg]

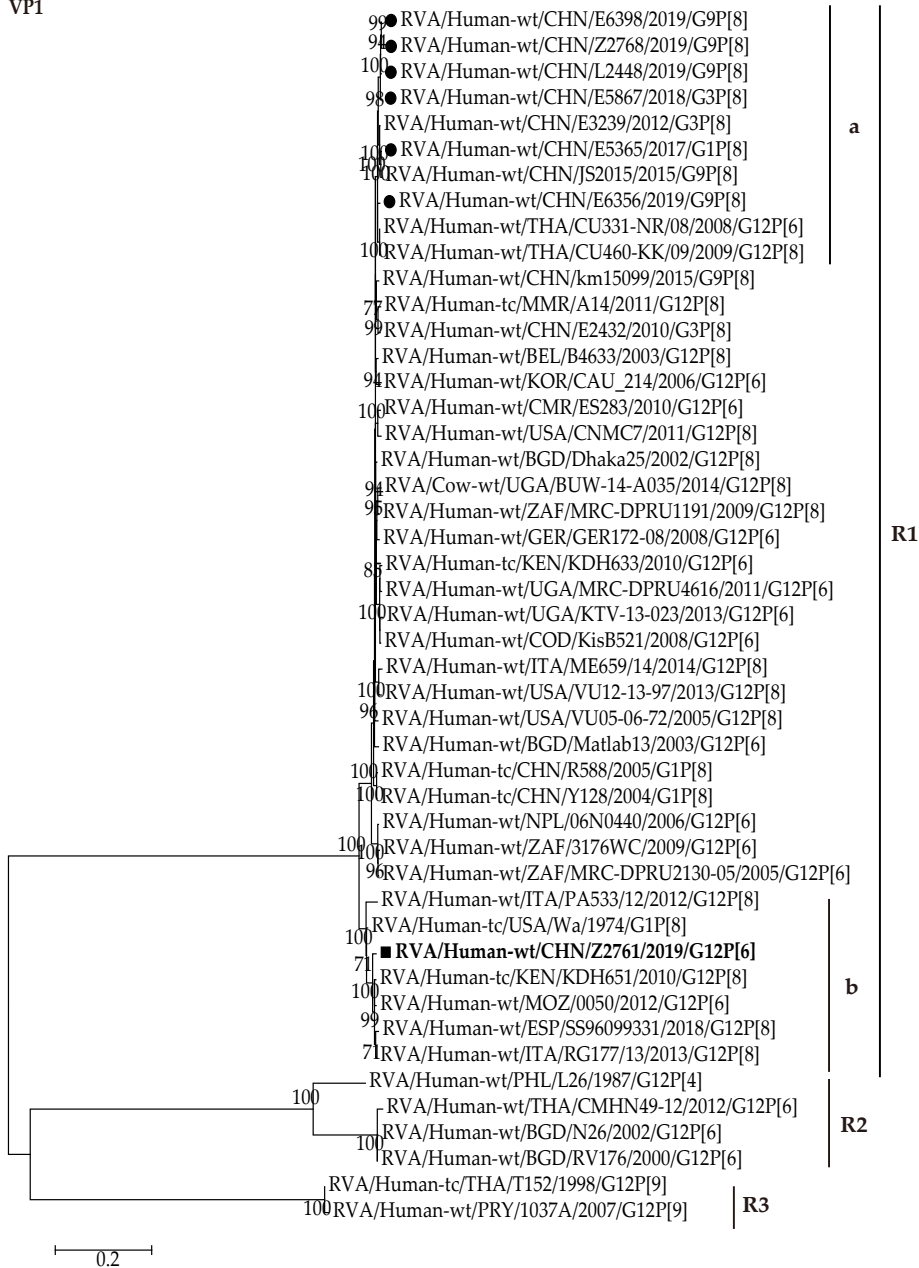

Supplement: Supplementary file 1 [file pathogens-09-00810-s001.zip › Supplementary Materials/Figure S2.pdf]

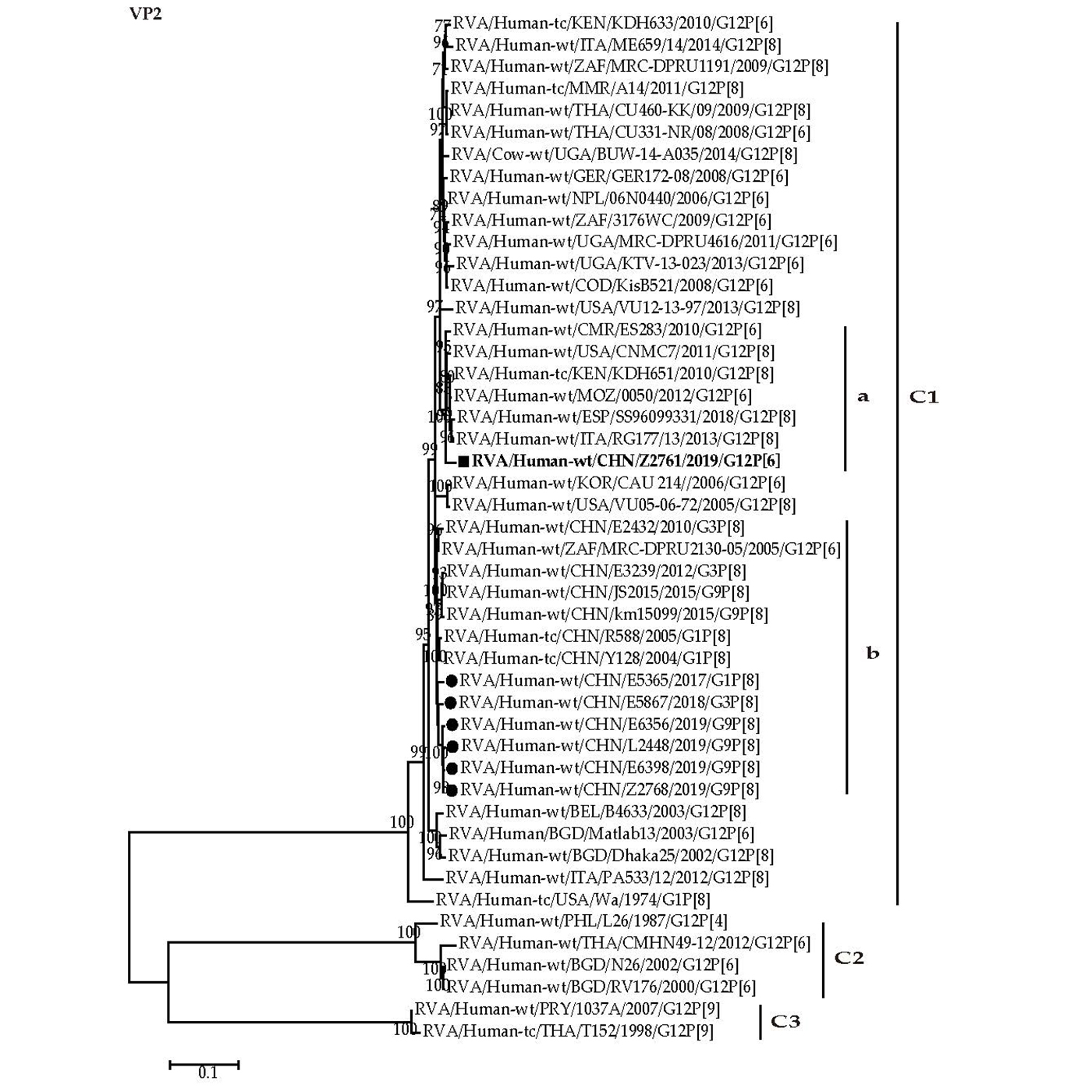

Supplement: Supplementary file 1 [file pathogens-09-00810-s001.zip › Supplementary Materials/Figure S3.jpg]

VP2

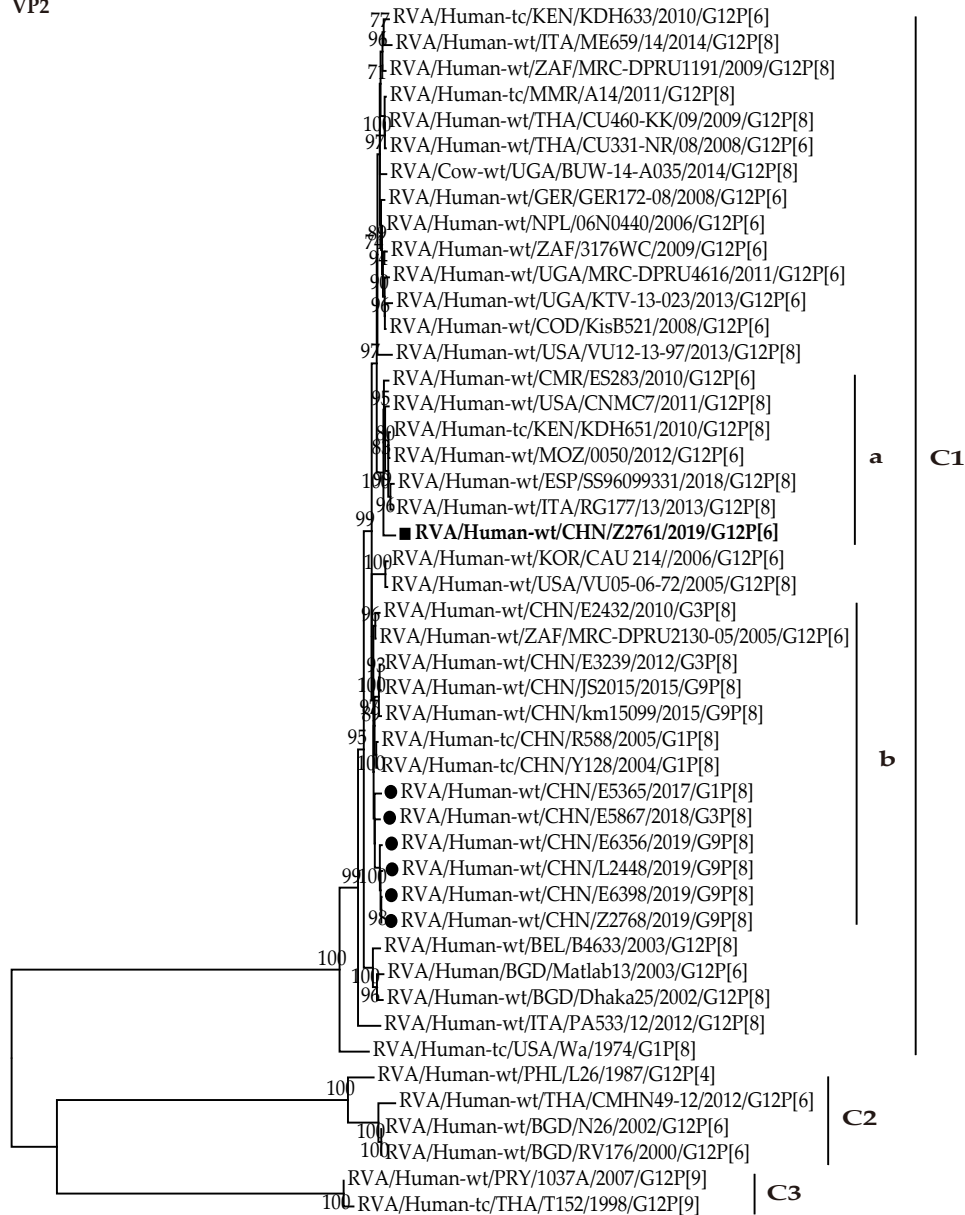

0.1

Supplement: Supplementary file 1 [file pathogens-09-00810-s001.zip › Supplementary Materials/Figure S3.pdf]

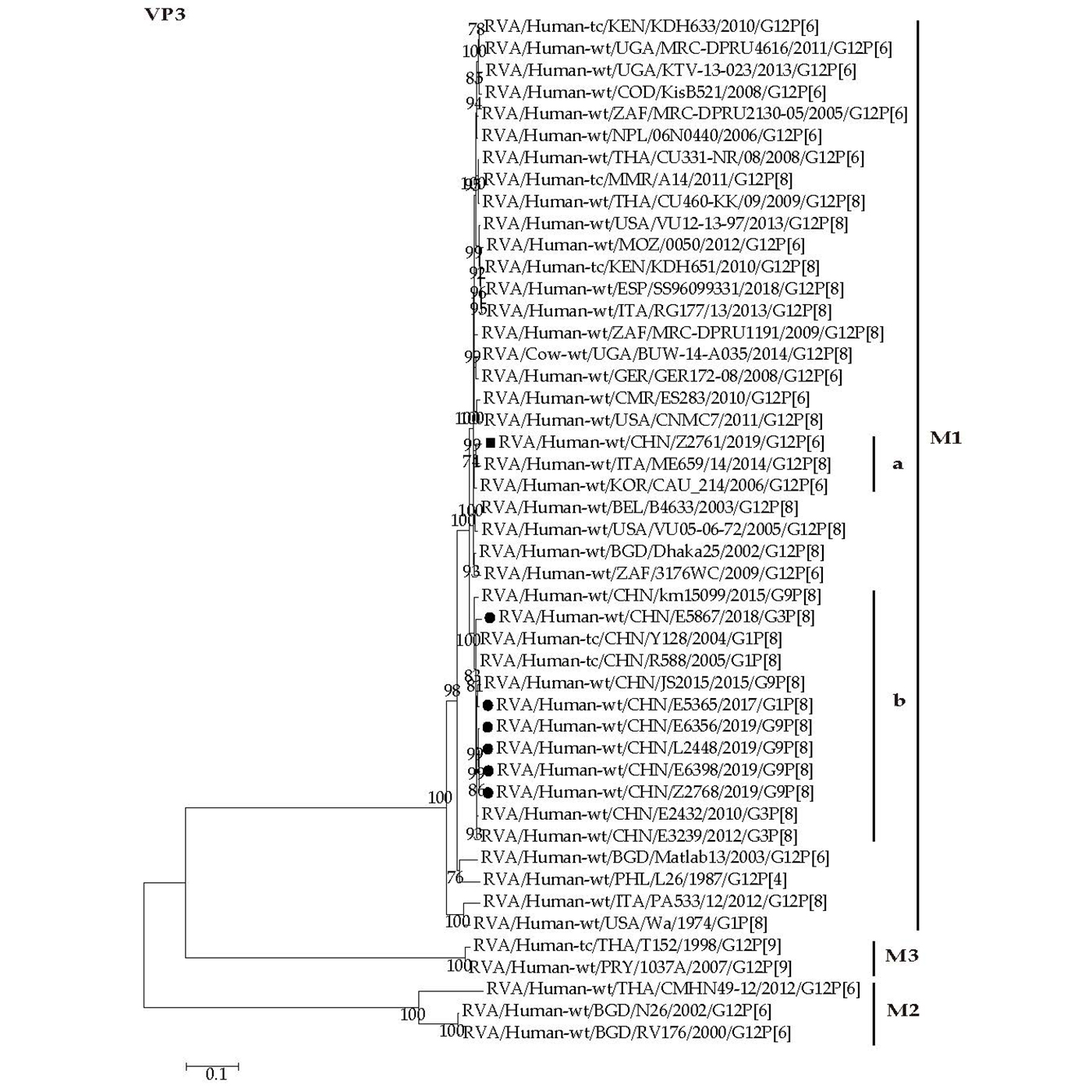

Supplement: Supplementary file 1 [file pathogens-09-00810-s001.zip › Supplementary Materials/Figure S4.jpg]

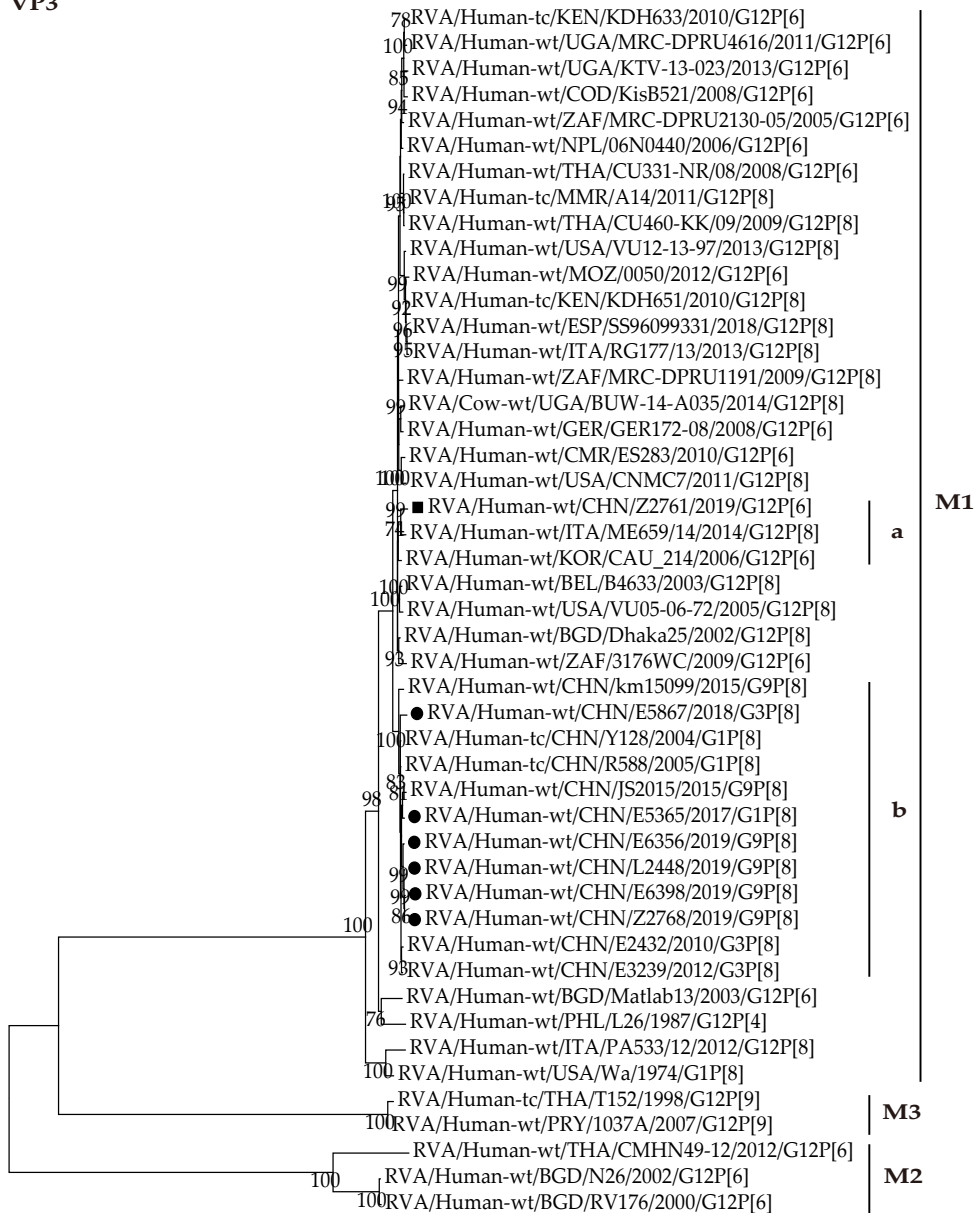

Supplement: Supplementary file 1 [file pathogens-09-00810-s001.zip › Supplementary Materials/Figure S4.pdf]

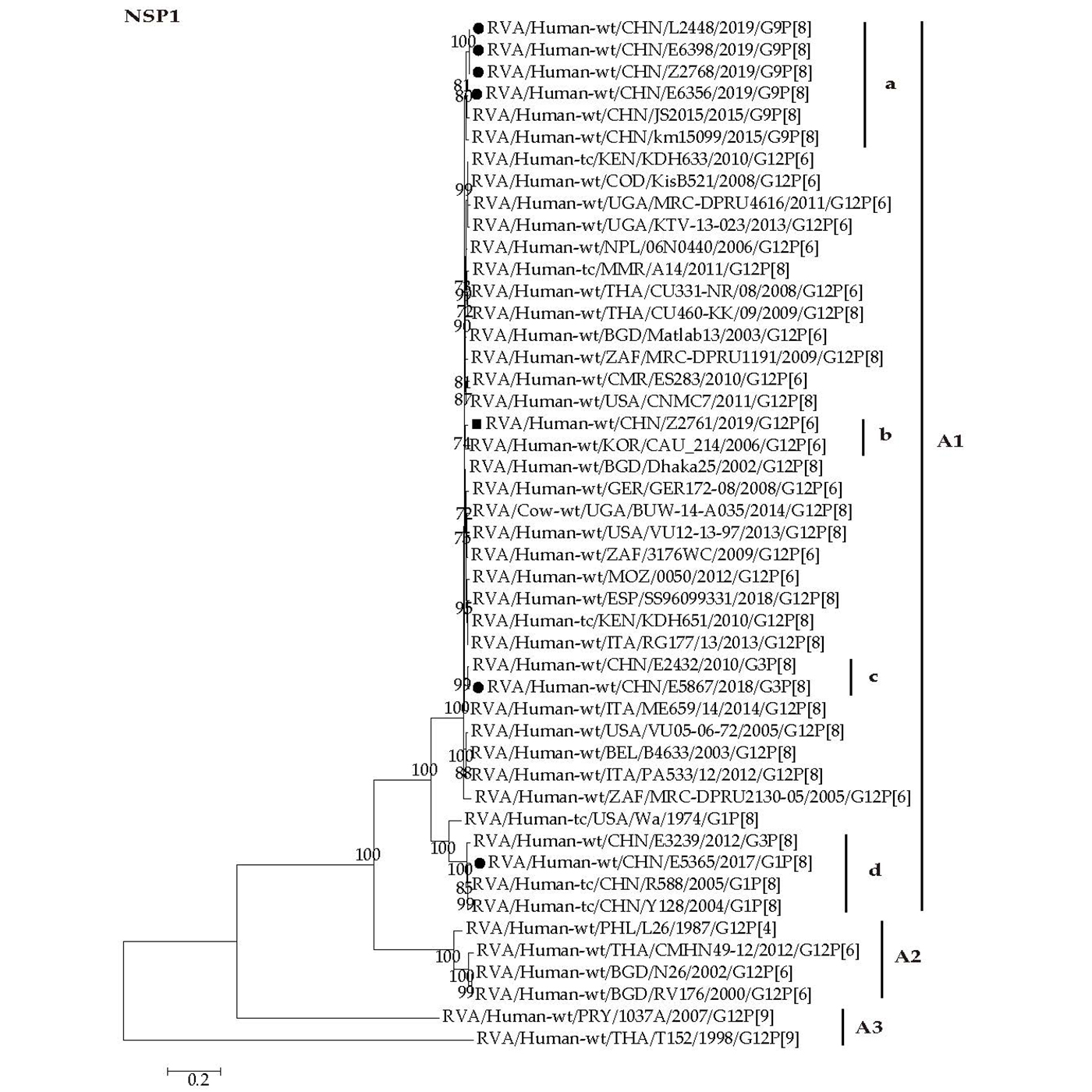

Supplement: Supplementary file 1 [file pathogens-09-00810-s001.zip › Supplementary Materials/Figure S5.jpg]

NSF 1

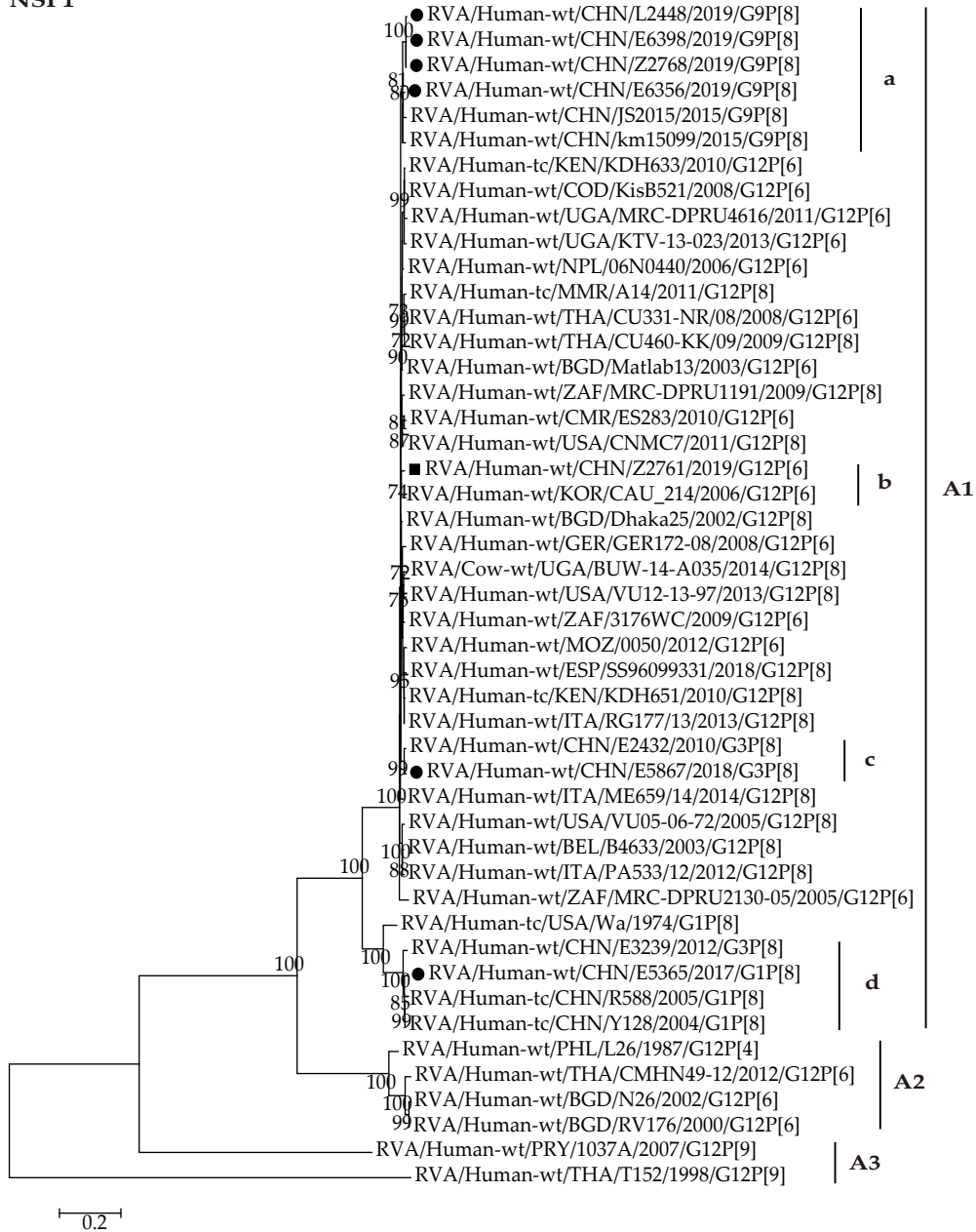

Supplement: Supplementary file 1 [file pathogens-09-00810-s001.zip › Supplementary Materials/Figure S5.pdf]

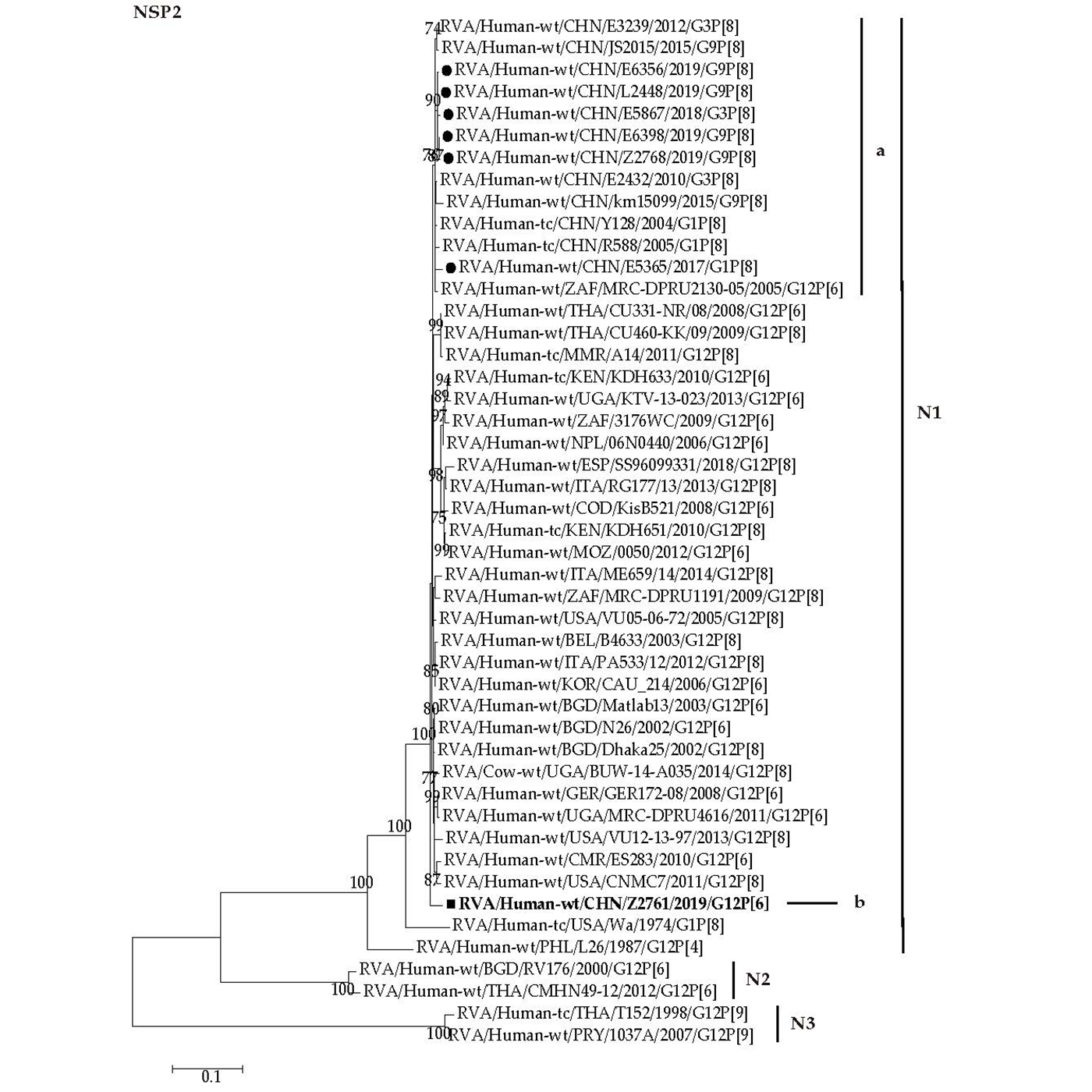

Supplement: Supplementary file 1 [file pathogens-09-00810-s001.zip › Supplementary Materials/Figure S6.jpg]

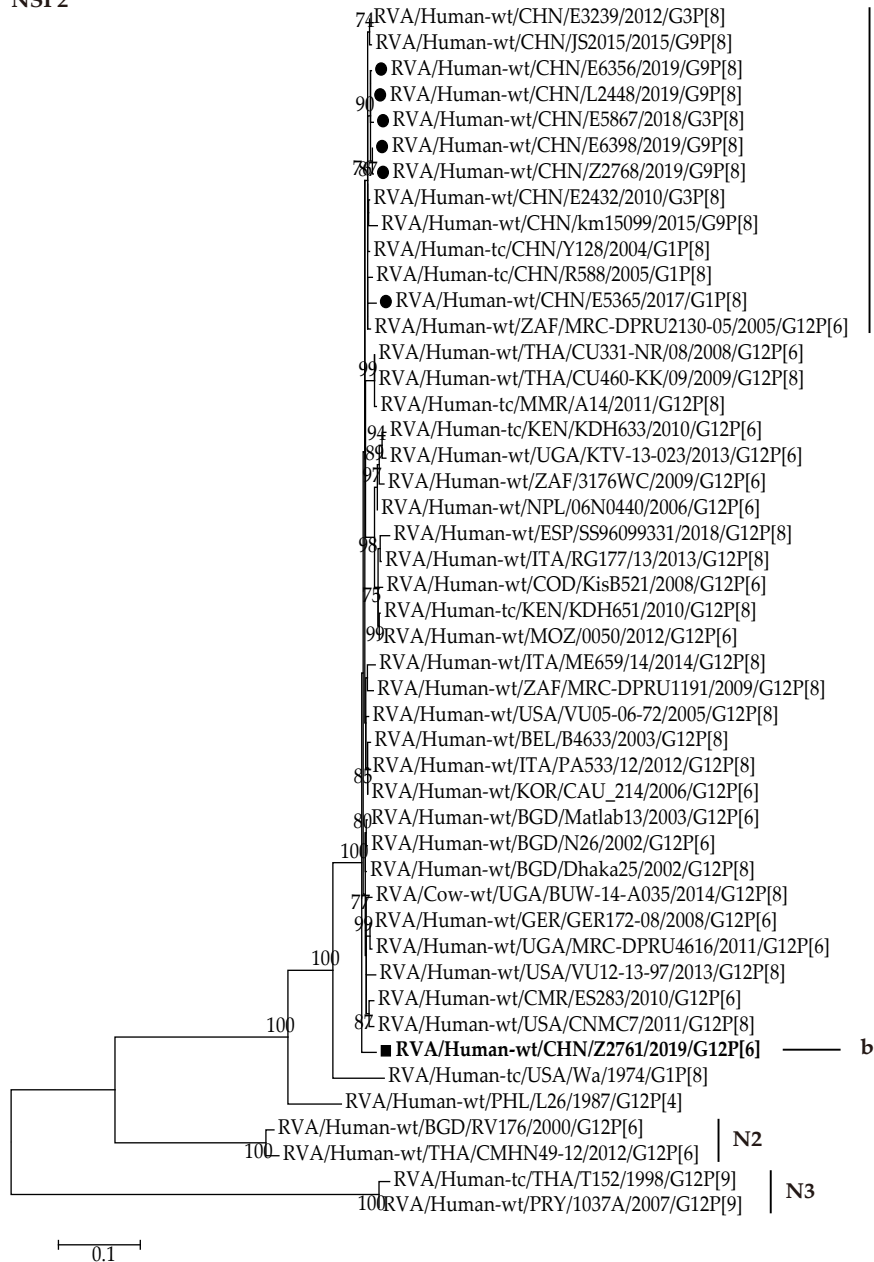

Supplement: Supplementary file 1 [file pathogens-09-00810-s001.zip › Supplementary Materials/Figure S6.pdf]

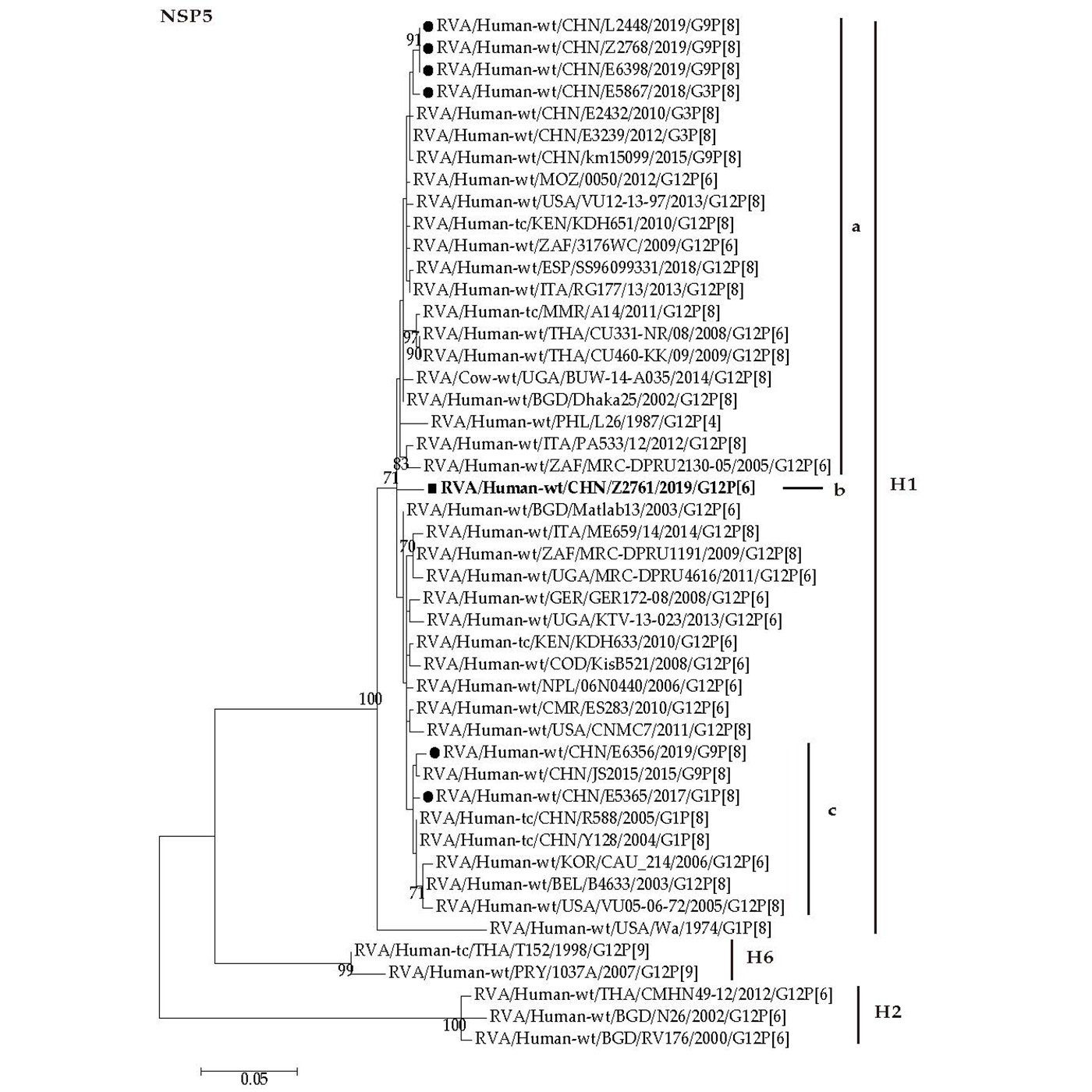

Supplement: Supplementary file 1 [file pathogens-09-00810-s001.zip › Supplementary Materials/Figure S7.jpg]

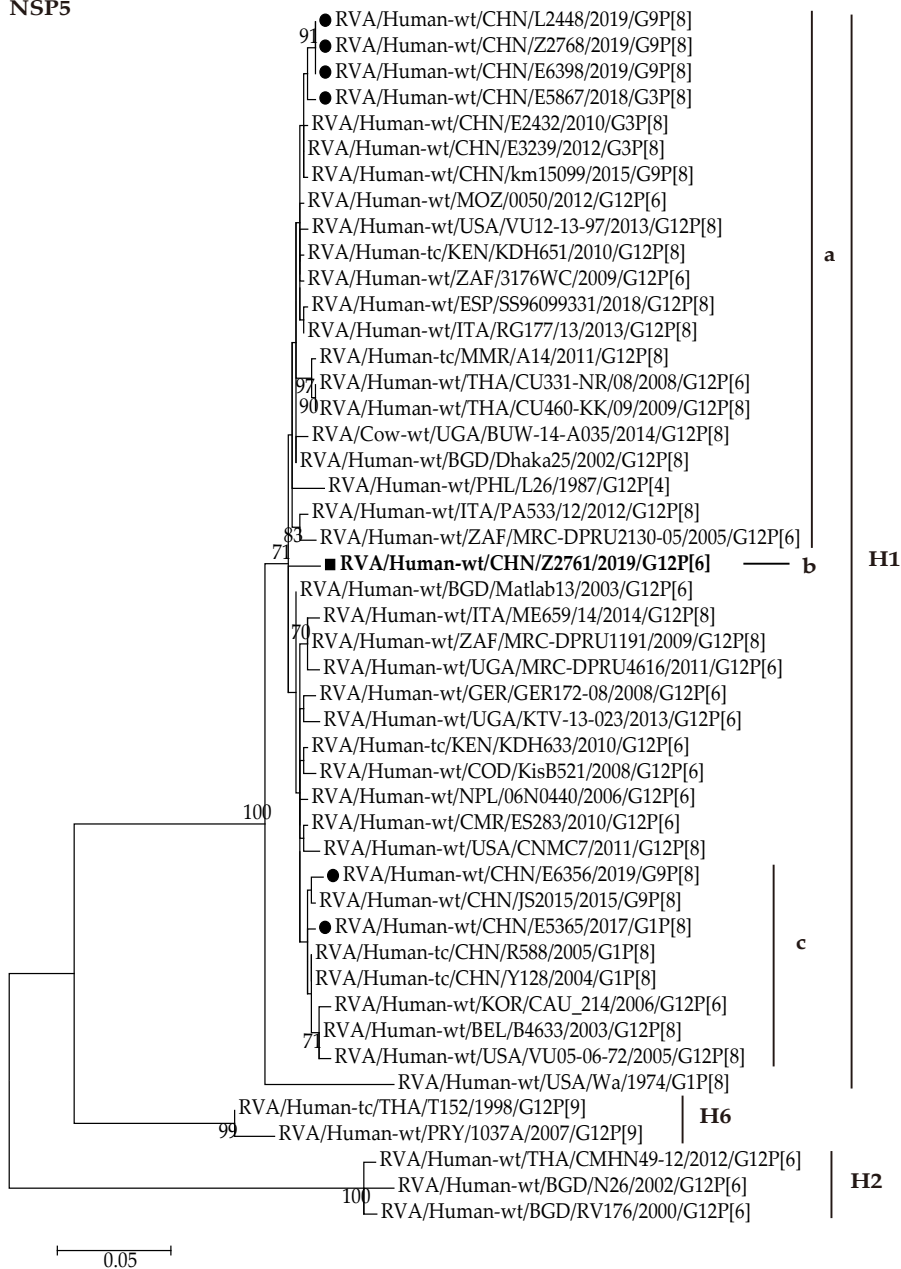

Supplement: Supplementary file 1 [file pathogens-09-00810-s001.zip › Supplementary Materials/Figure S7.pdf]
